# Draft Genome Sequences of Multiple *Frackibacter* Strains Isolated from Hydraulically Fractured Shale Environments

**DOI:** 10.1128/genomeA.00608-17

**Published:** 2017-08-10

**Authors:** Anne E. Booker, Michael D. Johnston, Rebecca A. Daly, Kelly C. Wrighton, Michael J. Wilkins

**Affiliations:** a Department of Microbiology, The Ohio State University, Columbus, Ohio, USA; b School of Earth Sciences, The Ohio State University, Columbus, Ohio, USA

## Abstract

The genomes of three novel *Frackibacter* strains (WG11, WG12, and WG13) were sequenced. These strains were isolated from hypersaline fluid collected from a hydraulically fractured natural gas well. These genomes provide information on the mechanisms necessary for growth in these environments and offer insight into interactions with other community members.

## GENOME ANNOUNCEMENT

Hydrocarbon-rich black shales underlie much of the continental United States and contain economically recoverable quantities of natural gas and oil (1). Hydraulic fracturing involves the high-pressure injection of water, chemical additives, and proppant into a shale formation, generating fracture networks that release oil and gas to be recovered at the surface (2). Three *Frackibacter* strains were isolated from fluid collected from a hydraulically fractured Utica shale well (3), indicating that this organism can persist despite the high temperatures (65°C), elevated pressures (5,000 psi), and dynamic salinities (20 to 170 g/liter total dissolved solids) characteristic of the deep shale environment (3, 4). *Frackibacter* is inferred to be an obligate fermenter capable of homoacetogenesis and glycine betaine and sugar fermentation (3). The 16S rRNA genes and whole-genomic sequences from these isolates (94% identity and 76% average nucleotide identity, respectively) are not closely related to any known organisms, indicating that *Frackibacter* may be native to hydraulically fractured shale environments (3). Here, we describe the genomic sequencing of three similar *Frackibacter* strains, WG11, WG12, and WG13, highlighting potential osmotic protection strategies.

*Frackibacter* isolates were grown in anaerobic yeast extract-peptone-dextrose (YPD) medium (ATCC medium 1245) at 40°C. Cells were harvested via centrifugation, and genomic DNA was isolated using a PowerSoil DNA isolation kit (Mo Bio). Genomic DNA was sequenced at the Department of Energy Joint Genome Institute (JGI), Walnut Creek, CA, USA. An Illumina shotgun library was constructed and sequenced using the HiSeq 2500-1 TB platform. The Illumina sequence data were assembled using the CLC Genomics Workbench (version 8.0.1) and AllPaths-LB (version r46652), generating 49, 54, and 51 contigs, which resulted in coverages of 570×, 518×, and 536× for WG11, WG12, and WG13, respectively. The G+C contents of strains WG11, WG12, and WG13 were 33.43%, 33.38%, and 33.39%, respectively. Genome annotation was performed via the Integrated Microbial Genomes platform (5) developed by the JGI and resulted in 2,693, 2,700, and 2,688 protein-coding genes, respectively. While all three strains are highly conserved (average nucleotide identity [ANI], 99.98%) at the genomic level, strain WG13 contains a type III clustered regularly interspaced short palindromic repeat (CRISPR)-associated (Cas) system and two CRISPR arrays of repeat and spacer sequences. Mechanisms for avoiding viral predation have previously been shown to be critical for persistence in terminal populations in hydraulically fractured shales (3).

Given that rapidly increasing salinity is a common geochemical trend across geographically distinct shale plays, mechanisms for halotolerance in shale-dwelling microbial populations are critical (4). We infer that *Frackibacter* strains WG11, WG12, and WG13 rely on the import of ions and compatible solutes for survival under elevated salinities. Each genome contained five *trkAH* genes involved in potassium ion uptake, and the compatible solutes are incorporated using multiple transporters (6). Each *Frackibacter* strain contains an integral membrane glycine betaine transporter and a three-component glycine betaine ABC transporter. Proline can enter the cell via this ABC transporter, in addition to a sodium/proline symporter, while betaine, carnitine, and choline can be imported into the cell by two separate transmembrane transporters (6). These novel isolate genomes will continue to be explored to gain insight into microbial subsurface survival strategies.

### Accession number(s).

These whole-genome shotgun sequences have been deposited in DDBJ/ENA/GenBank under the accession numbers FMZT01000001, FOCA01000001, and FOTT01000001 for WG11, WG12, and WG13, respectively. Additionally, these annotated genomes can be found at the JGI Integrated Microbial Genomes and Microbiome Samples under the IMG genome identification (ID) numbers 2642422545, 2642422556, and 2642422548, respectively.
